# Peripartum cardiomyopathy, what if your patient plans to reconceive?

**DOI:** 10.1002/ccr3.935

**Published:** 2017-04-04

**Authors:** Rashed Al Bannay, Aysha Husain, Zainab AlJufairi

**Affiliations:** ^1^ Cardiology unitSalmaniya Medical ComplexKingdom of Bahrain; ^2^ Obstetrics and GynecologySalmaniya Medical ComplexKingdom of Bahrain

**Keywords:** Dobutamine stress echocardiography, heart failure, peripartum cardiomyopathy, pregnancy

## Abstract

Patients with peripartum cardiomyopathy (PPCM) often express a desire to conceive again, and the risk of relapse in future pregnancies should be disclosed. No consensus is available that can determine that risk. Adequate contractile reserve, evidenced by a stress echocardiogram (exercise or dobutamine), can identify those with lower relapse risk.

## Introduction

Peripartum cardiomyopathy (PPCM) is myocardial disease of pregnancy in which heart failure develops during the last month of pregnancy or up to 5 months postdelivery with no identified cause. Patients might recover, but there is a risk of recurrence with subsequent pregnancies. Risk factors for recurrence are diverse and include clinical, biochemical, and echocardiographic variables. Dobutamine stress echocardiography (DSE) is reported in the literature to verify the recovery of the myocardium contractile reserve. We report two cases in which PPCM patients with recovered left ventricle (LV) function became pregnant again with no recurrence of their disease. These two females had a normal contractile reserve of LV with DSE prior to pregnancy, rendering them at low risk for recurrence.

## Case Report

### Case 1

A 21‐year‐old primigravida was diagnosed with PPCM after child birth. She was found to be short of breath 1 day postcesarean section delivery. Her pregnancy was uneventful, and she was not known to have any medical illnesses. She was hypotensive with a blood pressure (BP) of 90/60 mmHg and a heart rate of 140 beats per minute (bpm). She deteriorated rapidly and required endotracheal intubation, mechanical ventilation, and inotropic support in form of dopamine and dobutamine infusions. Despite the initial course, she recovered and was discharged home after optimization of her antifailure medications. Her medication regimen included B blocker (Bisoprolol), angiotensin converting enzyme inhibitors ACEi (Ramipril), and mineralocorticoid receptor blockers MRA (Spironolactone) that were titrated to maximum tolerated doses. Her six‐month echocardiography study showed a normalization of LV systolic function. One year later, she expressed her wish to become pregnant again. Her medications were tapered down with withdrawal of one medication at 2 weeks interval (first the furosemide, then spironolactone, then ramipril, and lastly the bisoprolol). To stratify her risk of PPCM recurrence, we elected to perform a DSE test to assess her myocardial recovery and contractile reserve.

### Case 2

A 22‐year‐old primigravida was diagnosed with PPCM after delivery of her child. She presented to the labor ward with shortness of breath and an elevated blood pressure of 180/110 mmHg. Her past medical history was unremarkable. She was initially managed as having pre‐eclampsia, but despite adequate blood pressure control and the delivery of her baby through cesarean section, she deteriorated further and required intubation and mechanical ventilation. Nitroglycerin infusion was used to control her blood pressure, and loop diuretic was added as decongestive therapy. B blockers (carvidelol), angiotensin receptor blockers ARB (Valsartan), and spironolactone were used and up titrated as tolerated. She progressively improved and was discharged after 10 days. Her LV systolic function has normalized by 6 months postdischarge. After 2 years, she and her husband were eager to have a second baby and wanted to know if it was going to be safe for her. We tapered down the medications with same scheme of previous patient. We performed DSE to judge the recovery of her myocardium and assess the contractile reserve after stopping all antifailure medications.

## Investigations

Table 1 shows the echocardiographic parameters at the time of diagnosis of PPCM and 6 months later. The systolic LV function on initial assessment as measured by Simpson's method was 40% in first patient and 20% in second patient. The global hypokinesia and the absence of apical ballooning made it unlikely to be stress‐related cardiomyopathy. Both females underwent DSE using a weight‐based infusion of dobutamine with 5 mcg per kg per min incremented every 5 min until reaching 85% of the age‐based target heart rate. Parasternal long axis, parasternal short axis, four‐chamber apical, and two‐chamber apical views were scanned at baseline, at the peak heart rate, and during recovery. The pulsed‐wave Doppler signal was used to trace the left ventricular outflow tract velocity time integral (LVOT VTI) for the calculation of stroke volume at each time point. Both patients showed normal LV function at baseline, with normal contractile reserve and an incremental increase in stroke volume by more than 20% during the dobutamine infusion, with no evidence of stress‐induced LV dysfunction. Table 2 shows the hemodynamic parameters of the DSE for the two patients. We had an extensive discussion with both couples. We told them that having a normal DSE might place the patients at a lower risk for a recurrence of PPCM; hence, the stress of dobutamine infusion mimics that of pregnancy. What was also conveyed that relapse cannot be totally ruled out, and close follow‐up is warranted.

**Table 1:** Echocardiographic parameters of the two patients at time of PPCM diagnosis and at 6 months after delivery

	Case 1		Case 2	
	Time of PPCM diagnosis	Six months post‐PPCM diagnosis	Time of PPCM diagnosis	Six months post‐PPCM diagnosis
LVIDD	53 mm	51 mm	54 mm	51 mm
LVIDS	42 mm	33 mm	46 mm	30 mm
EF %	40%	65%	20%	68%
E wave	89 m/sec	67 m/sec	87 m/sec	65 m/sec
A wave	21 m/sec	39 m/sec	23 m/sec	42 m/sec
DT	120 msec	165 msec	110 msec	160 msec

LVIDD, left ventricular dimension in diastole; LVIDS, left ventricular dimension in systole; EF, ejection fraction; DT, deceleration time.

**Table 2:** Hemodynamic data of the dobutamine stress test performed for the two patients after discontinuation of heart failure medication

	Case 1		Case 2	
	Baseline data	At maximum stress (85% of THR)	Baseline data	At maximum stress (85% of THR)
HR	70 bpm	170 bpm	76 bpm	172 bpm
BP	120/70 mmHg	140/80 mmHg	132/76 mmHg	138/80 mmHg
LVOT DM	2 cm	2 cm	2.3 cm	2.3 cm
LVOT VTI	19.5 cm	25.3 cm	20.3 cm	25.3 cm
SV	61 mL	76.3 mL	83 mL	105 mL
% of SV Increase		25%		26.5%

THR, target heart rate; HR, heart rate; BP, blood pressure; LVOT, left ventricle outflow tract; DM, diameter; VTI, velocity time integral; SV, stroke volume.

## Treatment and Follow‐up

### Case 1

The patient became pregnant again 4 months after her DSE. Serial follow‐up visits during her pregnancy revealed no evidence of symptoms suggestive of a PPCM recurrence. Echocardiography was repeated during her second trimester and again prior to delivery, and normal LV systolic function was found (Table 3). Her delivery was cesarean section, which proceeded normally with no complications. The family was pleased to have a second child with the mother remained healthy.

**Table 3:** Echocardiographic data for two patients during second trimester of the subsequent pregnancy**.**

	Case 1	Case 2
LVIDD	54 mm	52 mm
LVIDS	33 mm	31 mm
EF %	60%	65%
E wave	98 m/sec	100 m/sec
A wave	54 m/sec	58 m/sec
DT	164 msec	163 msec

LVIDD, left ventricular dimension in diastole; LVIDS, left ventricular dimension in systole; EF, ejection fraction; DT, deceleration time.

### Case 2

The patient decided to become pregnant again after she was stratified as low risk for recurrence of PPCM. She had follow‐up visits during her pregnancy with no evidence of heart failure symptoms. Her LV function was normal on echocardiography studies performed during her second trimester and prior to delivery (Table 3). She gave birth through a cesarean section delivery to a baby girl with no complications. The parents were grateful to have a second child in their family without experiencing the agony of the previous pregnancy.

## Discussion

We presented the clinical history of two females who recovered from PPCM and completed successful pregnancies after a normal DSE. How we estimate the risk of PPCM relapse in future pregnancies is a vexing question. Different markers have been identified that can negatively or positively predict myocardial recovery. Their variability reflects the ambiguity of PPCM etiology. These factors are diverse from being clinical, hormonal, or imaging modalities. Some examples include race, comorbidities, relaxin level, pro‐BNP titer, LV dimension in diastole, and global longitudinal strain 1, 2, 3, 4, 5. A consensus on how to estimate the risk of PPCM relapse is lacking. Contractile reserve may be an alternative worth exploring. Contractile reserve is defined as the difference between baseline and stress (both pharmacological and exercise) value of LV function 6. It is a parameter of prognostic significance for various heart failure syndromes and can be seen as a surrogate for recoverability 7, 8, 9. An example for the DSE utility in assessing the contractile reserve would be a state of low flow, low ejection fraction aortic stenosis 10. For PPCM, various reports have addressed the impact of contractile reserve on the prognosis. Normal contractile reserve, as demonstrated by a (dobutamine or exercise) echocardiogram, seems to be associated with a favorable outcome in subsequent pregnancies 11, 12.

Conflicting opinions exist as to whether dobutamine or exercise stress echocardiogram would be a best surrogate of the hemodynamics during pregnancy. PPCM is a heart failure syndrome confined to late pregnancy and the postpartum period 13. In mid‐to‐late pregnancy, cardiac output is maintained, primarily due to an increase in heart rate and a decrease in afterload. The latter is mediated through reduced total vascular resistance 14, 15. An elevated heart rate and reduced peripheral vascular resistance are similarly induced by a dobutamine infusion 16. However, exercise is known to increase systemic vascular resistance. In that respect, DSE simulates the normal physiology of pregnancy better than an exercise. However, the use of dobutamine as a pharmacological stress agent is reported to be associated with adverse event in one of each 300 tests 17. Ventricular arrhythmias are the most critical one 17. The probability of PPCM relapse during upcoming pregnancies will remain an uncertain estimate. There is no guarantee that heart failure will not occur in subsequent pregnancies, but normal contractile reserve can identify those with lesser risk. Will stress echocardiography become a key diagnostic for post‐PPCM patients considering a future pregnancy? More evidence is needed.

## Authorship

RAB: was a cardiology consultant involved in the case management, literature review, discussion writing, and final review of cases submitted. AH: was a cardiology resident involved in the case management, literature review, and case history writing. ZA: was a gynecology consultant involved in the case management, and literature review.

## Conflict of Interest

None declared.
